# Cell-specific delivery of CRISPR-Cas9 with pseudotyped lentiviral particles: Just change the envelope

**DOI:** 10.1016/j.omtn.2024.102395

**Published:** 2024-11-27

**Authors:** Ángela Covo-Vergara, Laura Salaberry, Noelia Silva-Pilipich, Sandra Hervas-Stubbs, Cristian Smerdou

**Affiliations:** 1 Division of Cancer, Cima Universidad de Navarra, Av. Pío XII 55, 31008 Pamplona, Spain; 2 Division of DNA and RNA Medicine, Cima Universidad de Navarra, Av. Pío XII 55, 31008 Pamplona, Spain; 3 Instituto de Investigación Sanitaria de Navarra (IdISNA) and CCUN, Pamplona, Spain; 4 Nanogrow Biotech, Montevideo, Uruguay

## Main text

The manuscript by Nielsen et al. describes a strategy based on pseudotyping lentivirus-derived nanoparticles (LVNPs) to deliver CRISPR-Cas9 ribonucleoprotein (RNP) complexes for cell-specific gene editing.^1^ This approach allowed the authors to efficiently edit several genes in specific cell types by pseudotyping LVNPs with envelope proteins from three different viruses, including SARS-CoV-2, Nipah virus, and measles virus. The importance of this study lies in its demonstration that LVNPs can be readily tuned to target different cell types, which could potentially enable gene editing *in vivo*, although the experiments shown in this work were only performed in cell culture.

Genome editing with CRISPR-Cas9 has been shown to be an extremely useful research tool with great therapeutic potential. Recently, the FDA approved an *ex vivo* gene editing therapy for β-thalassemia and sickle cell disease, known as Casgevy.^2^ This therapy uses CRISPR-Cas9 to knock out the *BCL11A* gene, a strong repressor of fetal γ-globin expression in adults. This editing approach restores γ-globin production in patients with thalassemia, compensating for their β-globin deficiency and reestablishing the production of functional hemoglobin. Interestingly, in Casgevy, CRISPR-Cas9 RNPs are delivered into patient hematopoietic stem cells via electroporation, a technique that lacks cell type specificity. Since viruses have evolved over millions of years to efficiently infect specific cells, the authors reasoned that they could serve as optimal vehicles for cell-specific gene editing. In fact, they had previously reported that LVNPs pseudotyped with vesicular stomatitis virus G protein (VSV-G) were able to incorporate and deliver RNPs containing base editors and prime editors.^3^

The incorporation of RNPs inside lentiviral and retroviral particles is possible thanks to the intrinsic property of the Gag protein to self-oligomerize and bud, embedding membrane-anchored proteins in the envelope of newly formed particles, even in the absence of viral RNA. Several studies have shown that these particles can also include heterologous proteins, such as Cas9, by fusing them to Gag with a protease-cleavable linker, enabling their release after cell transduction.^4^^,^^5^ Pseudotyping of LVNPs can be achieved when Gag and a viral envelope protein are coexpressed in the same packaging cells.

**Figure 1:**
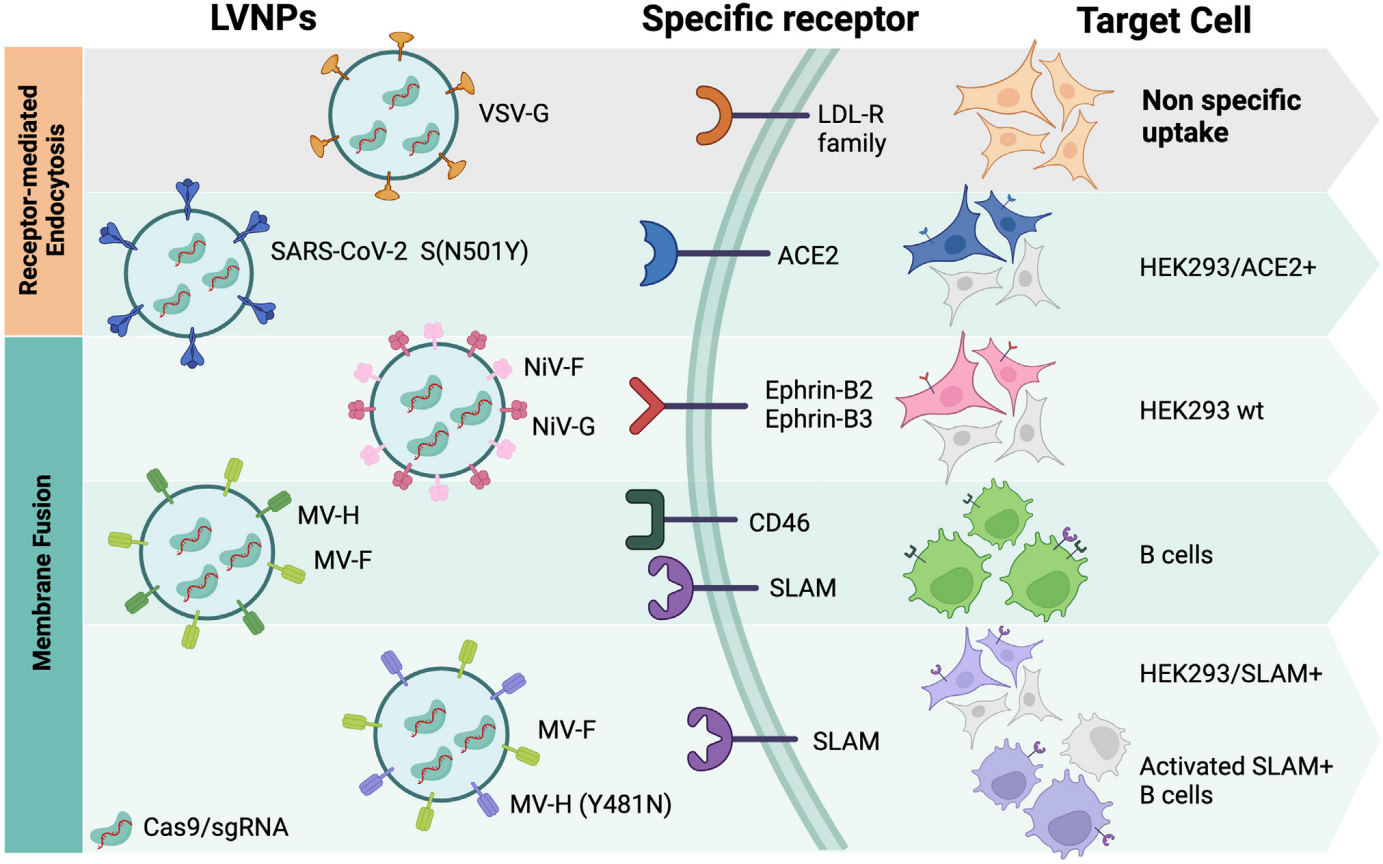
Targeting LVNPs carrying CRISPR-Cas9 to specific cells by pseudotyping with different viral proteins ACE2, angiotensin-converting enzyme 2; LDL-R, low-density lipoprotein receptor; MV-F and MV-H, measles virus fusion and hemagglutinin proteins, respectively; NiV-F and NiV-G, Nipah virus fusion and attachment proteins, respectively; sgRNA, single guide RNA; SLAM, signaling lymphocytic activation molecule; VSV-G, vesicular stomatitis virus G protein; WT, wild type. This figure was created with Biorender.com.

Nielsen et al. demonstrated that LNVPs pseudotyped with VSV-G, a protein commonly used for pseudotyping lentivirus vectors, achieved high transduction efficacy, but exhibited broad cell tropism, due to its binding to the low-density lipoprotein receptor. which is widely expressed across many cell types.^1^ To increase the specificity of this system, they decided to pseudotype LVNPs with envelope proteins from viruses with different tropisms. For this purpose, they chose viruses that can enter cells through two different routes: endosome escape, such as SARS-CoV-2, and membrane fusion, such as Nipah and measles viruses (Figure 1). In the first case, they showed that LVNPs pseudotyped with the SARS-CoV-2 spike protein (mutant N501Y) and loaded with RNPs targeting the *AFF1* gene led to 100% insertion or deletion (indel) formation only in cells expressing the angiotensin-converting enzyme 2 (ACE2), which is the natural viral receptor. One potential application of ACE2-specific LVNPs is delivering RNPs to lung epithelial cells to correct *CFTR* gene variants responsible for cystic fibrosis, as the ACE2 receptor is highly expressed in this tissue.

On the other hand, the flexibility of this system was also demonstrated with LVNPs pseudotyped with envelope proteins mediating fusion at the plasma membrane. One example includes LVNPs pseudotyped with Nipah virus glycoproteins NiV-G (attachment protein) and NiV-F (fusion protein), which can mediate viral uptake by binding to Ephrin-B2 or Ephrin-B3 receptors (EphB3/4R). NiV-G/F-pseudotyped LVNPs exhibited a strong specificity for HEK293T cells expressing EphB3/4R, leading to a knockout of 70% of the *B2M* gene when loaded with RNPs targeting this gene. However, no editing was observed with these LVNPs in EphB3/4R-deficient cells, while VSV-G-pseudotyped LNVPs were equally efficient at transducing and editing cells regardless of EphB3/4R expression. This specific targeting could be particularly relevant for genome editing in endothelial cells, with potential applications for treating cardiovascular diseases.^6^

Finally, the authors also explored the use of measles virus glycoproteins for targeted delivery to B lymphocytes. To achieve this, they pseudotyped LNVPs with measles virus fusion (MV-F) and hemagglutinin (MV-H) proteins. This latter protein enables targeting of immune cells, including T and B cells, due to its ability to interact with the signaling lymphocytic activation molecule (SLAM) present on their surface. However, since MV-H also has the capacity to bind the more widely expressed CD46 membrane protein, a mutant version (Y481N) was also tested. This mutant was able to confer LNVPs a very specific targeting to SLAM^+^ cells, such as activated B cells, in which >80% of *AFF1* gene editing was achieved. Interestingly, VSV-G-pseudotyped LVNPs were very inefficient at targeting B cells. A potential application of B cell engineering could be the *in vivo* expression of specific antibodies.^7^

These results highlight the versatility of LVNPs, demonstrating that they can be tailored with different viral envelope proteins to selectively target specific cell subpopulations, potentially enabling gene editing *in vivo*. The fact that the system only delivers the RNP complex to the target cell endows it with a high degree of safety, as this complex will have a short life inside the cells, reducing the risk for off-target editing. Despite its potential, a possible limitation of this technology, as noted by the authors, is the likely production of neutralizing antibodies against the viral proteins used for pseudotyping. This could restrict its use in individuals with pre-existing immunity or limit its ability to be re-administered. However, the versatility of the system could allow LVNPs to be pseudotyped with different serotypes of the same viral protein, potentially reducing the impact of neutralizing antibodies and allowing, in some cases, their re-administration *in vivo*. An additional limitation of the system is that it can only be used for gene knockout, relying on non-homologous end joining. Knocking in strategies would need to simultaneously provide a DNA repair template to facilitate homologous recombination. Although this could be achieved by providing the DNA sequence with another viral vector, such as adeno-associated virus, or by transfection with a plasmid, it would certainly decrease the efficacy and specificity of this system. An attractive alternative approach could be to develop LNVPs engineered to also co-package DNA or an RNA that could be retrotranscribed to cDNA without being integrated into the genome of target cells.^8^ In any case, the pseudotyped LVNPs described in this work contribute to the increasing arsenal of gene editing tools and will likely facilitate the clinical translation of these therapies, especially in those cases where cell-specific targeting is required.

## Acknowledgments

This work is funded by the following grants to C.S.: project PI23/00565 funded by Instituto de Salud Carlos III (ISCIII) and co-funded by the European Union, Gobierno de Navarra, Departamento de Salud (GN2022/21), and Fundación Intheos.

## Declaration of interests

L.S. and N.S.-P. are currently employees of Nanogrow Biotech.
